# Microbiological profile and antibiotic susceptibility profile of urine cultures in patients with spinal cord injury—retrospective study

**DOI:** 10.1097/j.pbj.0000000000000272

**Published:** 2024-11-14

**Authors:** Nuno J. S. Ferreira, Raquel A. Branco, Sabrina C. Pimentel, Maria Ana S. Paço, Isabel M. S. R. Coelho, Lúcia E. P. R. Serpa

**Affiliations:** a Laboratory of Clinical Microbiology, Department of Clinical Pathology, Hospital do Divino Espírito Santo, Ponta Delgada, S. Miguel—Azores, Portugal; b Department of Physical and Rehabilitation Medicine, Hospital do Divino Espírito Santo, Ponta Delgada, S. Miguel—Azores, Portugal

**Keywords:** microbiological profile, urine cultures, spinal cord injury

## Abstract

**Background:**

Urinary tract infections (UTIs) and urinary tract colonizations (UTCs) are common in patients with spinal cord injury (SCI). The aim of this study was to characterize the microbiological profile of urine cultures in patients with SCI and to determine the antibiotic susceptibility profile of most common microorganisms, to track antibiotic resistance and facilitate empiric antibiotic selection.

**Methods:**

A retrospective observational study was conducted on 235 urine culture results of 29 patients with SCI followed at a Physical and Rehabilitation Medicine outpatient consultation between January 2016 and April 2024. Data regarding sociodemographics, cause of SCI, American Spinal Injury Association Impairment Scale classification, voiding method, microbiological urine culture profile, and antimicrobial resistance were collected and statistically analyzed. UTIs (defined as bacteriuria, leukocyturia, positive urine culture, and new onset of signs and/or symptoms) were differentiated from UTCs.

**Results:**

Patients were mostly men (86%), with a mean age of 52.1 years. UTIs occurred in 134 specimens (57%) and UTCs in 101 (43%). In both UTIs and UTCs, microbiological agents were mostly bacteria; *Escherichia coli* was the commonest overall (39%) and more frequent in indwelling catheterization (in UTIs) and intermittent self-catheterization (in UTCs); more frequently identified microorganisms were *E. coli*, *Klebsiella pneumoniae*, *Pseudomonas aeruginosa*, *Proteus mirabilis*, and *Enterococcus faecalis*. For these 5 more frequent bacteria, antibiotic susceptibility profiles were determined. High resistance to fluoroquinolones, low resistance to cephalosporins, and very low resistance to nitrofurantoin were found. Specific multidrug-resistant organisms (MDROs) accounted for 11.2%, mostly identified in patients with indwelling catheters. Antibiotic prescriptions in UTIs were according to antibiograms.

**Conclusions:**

In UTIs and UTCs, *E. coli* was the most common microorganism; microorganisms were distinct on different types of voiding methods. Antibiotic susceptibility profiles were determined for the more frequent bacteria. Very low resistance to nitrofurantoin of *E. coli* and *E. faecalis*, low resistance to cephalosporins, and high resistance to fluoroquinolones were found. The data now reported can, in selected cases, facilitate empiric antibiotic selection.

## Introduction

The European Association of Urology (EAU), in its most recent guidelines to date, classifies urinary tract infections (UTIs) as uncomplicated (those occurring in nonpregnant women with no known relevant anatomical and functional abnormalities within the urinary tract and comorbidities) and complicated (all that are not defined as uncomplicated, including those occurring in men and patients with functional abnormalities of the urinary tract and/or indwelling urinary catheters).^1^ According to this classification, UTIs in patients with spinal cord injury (SCI) are considered complicated.

In patients with SCI, an UTI is characterized by new onset of signs and/or symptoms accompanied by laboratory findings (bacteriuria, leukocyturia, and positive urine culture).^2^ The signs and symptoms can differ from those in healthy individuals. Patients with SCI may have other signs and symptoms besides the traditional signs and symptoms of UTIs; some symptoms may be absent because of alterations in sensibility, and other problems may develop or worsen during UTIs.^3^ Therefore, they may have cloudy urine, malodorous urine, urinary incontinence/failure of control or leaking around the catheter, fever (or elevated body temperature in those prone to poikilothermia), autonomic dysreflexia, increased spasticity, kidney/bladder discomfort, back pain, feeling sick, malaise, lethargy, or sense of unease.

Bacteriuria, leukocyturia, and positive urine culture are the laboratory findings that support diagnosis of UTIs in symptomatic patients. Bacteriuria is defined as a count of colony-forming units (CFUs) of at least 10^3^/mL; leukocyturia is defined as ≥30 × 10^6^ leukocytes (white blood cells [WBCs]) per liter of urine (30 WBCs × 10^6^/L), with a count of 10–30 WBCs x 10^6^/L considered borderline^4^; a positive urine culture requires the growth of up to 2 distinguishable isolates belonging to species recognized as uropathogens.^4^

Asymptomatic bacteriuria is defined in the European Federation of Clinical Chemistry and Laboratory Medicine (EFLM) European Urinalysis Guideline as the presence of 1 or 2 species with growth at 10^5^ CFUs or more in a properly collected midstream specimen, regardless of leukocyturia, in the absence of signs and symptoms of UTI. It is a common occurrence (23–89% in patients^1^ with SCI and close to 100% in individuals with indwelling urinary catheters^4^) and corresponds to a commensal colonization of the urinary tract (urinary tract colonization [UTC])^1^. Both EFLM and EAU guidelines recommend that UTC must generally not be treated, with only a few exceptions, such as pregnant women or before invasive urologic procedures.^1,4^

Antimicrobial resistance is a major public health problem. Antibiograms are useful to track antibiotic resistance, to guide empiric treatment guidelines, and to facilitate empiric antibiotic selection.^5^

The aim of this study was to characterize the microbiological profile of urine cultures in patients with SCI and to determine the antibiotic susceptibility profile of most common microorganisms, to track antibiotic resistance and facilitate empiric antibiotic selection.

## Methods

This was a retrospective observational study of 235 urine culture results of 29 patients with SCI followed at a Physical and Rehabilitation Medicine (PRM) outpatient consultation of a district Portuguese hospital between January 2016 and April 2024. Data regarding sociodemographics, cause of SCI, ASIA (American Spinal Injury Association) Impairment Scale (AIS) classification, and voiding method, as well as microbiological urine culture profile and antimicrobial resistance data, were collected and statistically analyzed. Data collection and analysis were performed according to confidentiality and ethical principles (including the ethical standards of the mentioned hospital and the Declaration of Helsinki). Urinary tract infection (defined as bacteriuria, leukocyturia, positive urine culture, and new onset of signs and/or symptoms) was differentiated from urinary tract colonization.

In the Laboratory of Clinical Microbiology, urine cultures were performed applying routine internal protocols. Accurate microorganism identification and antimicrobial susceptibility testing were performed with the Vitek2 system (bioMérieux, Portugal) and interpreted according to current European Committee on Antimicrobial Susceptibility Testing (EUCAST) guidelines.^6^

## Results

Patients were mostly men (25 (86%)), with a mean age of 52.1 years; 15 (51.7%) were retired. Etiology of SCI, AIS classification, and voiding methods are presented in Table 1.

**Table 1:** Etiology of SCI, AIS classification, and voiding methods.

	N (%)
Etiology of SCI	
Traumatic	22 (75.9%)
Fall	11 (37.9%)
Car accident	8 (27.7%)
Diving accident	3 (10.4%)
Inflammatory	2 (6.9%)
Iatrogenic	2 (6.9%)
Compressive	1 (3.4%)
Infectious	1 (3.4%)
Neoplastic	1 (3.4%)
AIS classification	
Tetraplegia	13 (44.8%)
AIS A	4 (13.8%)
AIS C	2 (6.9%)
AIS D	7 (24.1%)
Paraplegia	16 (55.2%)
AIS A	9 (31%)
AIS B	3 (10.4%)
AIS C	1 (3.4%)
AIS D	3 (10.4%)
Voiding method	
Intermittent self-catheterization	11 (37.9%)
Intermittent catheterization	1 (3.4%)
Indwelling catheterization	7 (24.2%)
Suprapubic catheterization	1 (3.4%)
Condom catheterization	1 (3.4%)
Spontaneous voiding	8 (27.7%)

ASIA, American Spinal Injury Association; AIS, ASIA Impairment Scale; N, number; SCI, spinal cord injury.

UTIs occurred in 134 urine cultures (57%); UTCs occurred in 101 (43%). *E. coli* was the most common microorganism overall (39%).

In both UTIs and UTCs, microbiological agents were mostly bacteria (99.3% and 98.1%, respectively); *E. coli* was the most common microorganism (40% and 36.9%, respectively) and was more frequent in patients who do indwelling catheterization (in UTIs) and intermittent self-catheterization (in UTCs).

**Figure 1.**
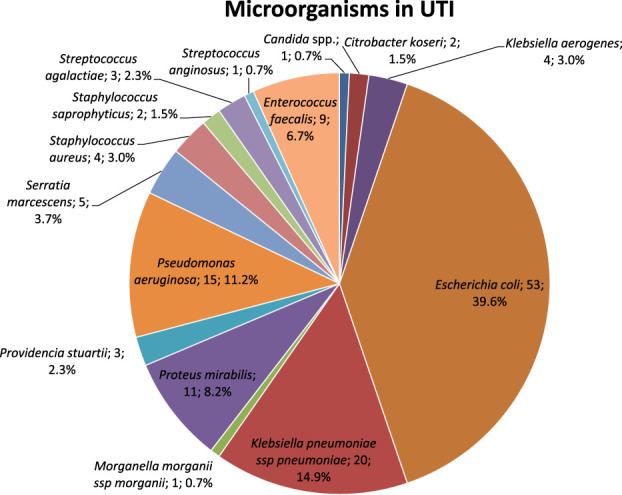
Microorganisms identified in UTIs.

In UTIs, identified microorganisms included the following (Fig. 1): *E. coli* 39.6%, *K. pneumoniae* 14.9%, *P. aeruginosa* 11.2%, *P. mirabilis* 8.2%, *E. faecalis* 6.7%, *Serratia marcescens* 3.7%, *Klebsiella aerogenes* 3.0%, *Staphylococcus aureus* 3.0%, *Providencia stuartii* 2.3%, *Streptococcus agalactiae* 2.3%, *Citrobacter koseri* 1.5%, *Staphylococcus saprophyticus* 1.5%, *Candida* spp. 0.7%, *Morganella morganii* 0.7%, and *Streptococcus anginosus* 0.7%.

Distinguishing by voiding methods, microorganisms causing UTIs included the following: in intermittent self-catheterization, *E. coli* 60.6%, *K. pneumoniae* 21.2%, *S. marcescens* 6.1%, *S. saprophyticus* 6.1%, *C. koseri* 3.0%, and *S. aureus* 3.0%; in intermittent catheterization, *P. mirabilis* 36.3%, *K. pneumoniae* 27.3%, *E. faecalis* 13.6%, *S. agalactiae* 13.6%, *M. morganii* 4.6%, and *S. marcescens* 4.6%; in indwelling catheterization, *E. coli* 39.7%, *P. aeruginosa* 17.3%, *E. faecalis* 8.7%, *K. pneumoniae* 8.6%, *K. aerogenes* 6.9%, *P. stuartii* 5.2%, *P. mirabilis* 3.4%, *S. marcescens* 3.4%, *S. aureus* 3.4%, *Candida* spp. 1.7%, and *S. anginosus* 1.7%; in suprapubic catheterization, *E. faecalis* 33.4%, *K. pneumoniae* 33.3%, and *S. aureus* 33.3%; in condom catheterization, *E. coli* 100%; and in spontaneous voiding, *E. coli* 46.6%, *P. aeruginosa* 33.3%, *C. koseri* 6.7%, *K. pneumoniae* 6.7%, and *P. mirabilis* 6.7%, as summarized in Table 2.

**Table 2:** Microorganisms by the voiding method in UTIs.

	Intermittent self-catheterization		Intermittent catheterization		Indwelling catheterization		Suprapubic catheterization		Condom catheterization		Spontaneous voiding		Total	
	N	%	N	%	N	%	N	%	N	%	N	%	N	%
*Candida* spp.					1	1.7%							1	0.7%
*Citrobacter koseri*	1	3.0%									1	6.7%	2	1.5%
*Klebsiella aerogenes*					4	6.9%							4	3.0%
*Enterobacter cloacae complex*														
*Escherichia coli*	20	60.6%			23	39.7%			3	100%	7	46.6%	53	39.6%
*Klebsiella oxytoca*														
*Klebsiella pneumoniae ssp pneumonia*	7	21.2%	6	27.3%	5	8.7%	1	33.3%			1	6.7%	20	14.9%
*Morganella morganii ssp morganii*			1	4.6%									1	0.7%
*Proteus mirabilis*			8	36.3%	2	3.4%					1	6.7%	11	8.2%
*Providencia stuartii*					3	5.2%							3	2.3%
*Pseudomonas aeruginosa*					10	17.3%					5	33.3%	15	11.2%
*Serratia marcescens*	2	6.1%	1	4.6%	2	3.4%							5	3.7%
*Staphylococcus aureus*	1	3.0%			2	3.4%	1	33.3%					4	3.0%
*Staphylococcus saprophyticus*	2	6.1%											2	1.5%
*Streptococcus agalactiae*			3	13.6%									3	2.3%
*Streptococcus anginosus*					1	1.7%							1	0.7%
*Enterococcus faecalis*			3	13.6%	5	8.6%	1	33.4%					9	6.7%
													134	100%

N, number.

**Figure 2.**
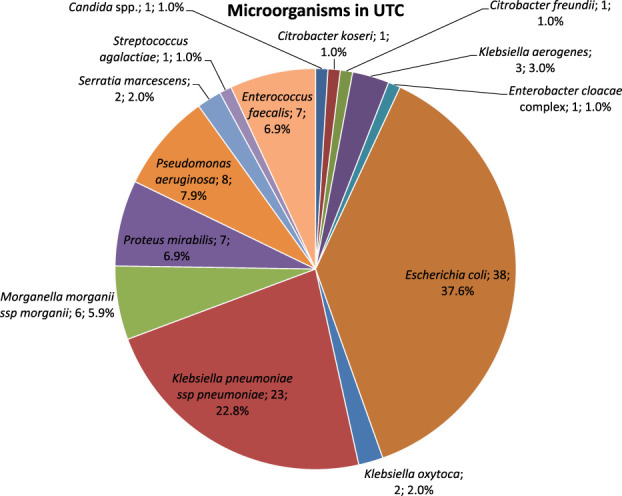
Microorganisms identified in UTCs.

In UTCs, identified microorganisms included the following (Fig. 2): *E. coli* 37.6%, *K. pneumoniae* 22.8%, *P. aeruginosa* 7.9%, *E. faecalis* 6.9%, *P. mirabilis* 6.9%, *M. morganii* 5.9%, *K. aerogenes* 3.0%, *K. oxytoca* 2.0%, *S. marcescens* 2.0%, Candida spp. 1.0%, *C. freundii* 1.0%, *C. koseri* 1.0%, *E. cloacae* 1.0%, and *S. agalactiae* 1.0%.

Distinguishing by voiding methods, microorganisms identified in UTCs included the following: in intermittent self-catheterization, *E. coli* 53.3%, *K. pneumoniae* 29%, *P. aeruginosa* 4.5%, *C. freundii* 2.2%, *C. koseri* 2.2%, *K. aerogenes* 2.2%, *K. oxytoca* 2.2%, *M. morganii* 2.2%, and *S. agalactiae* 2.2%; in intermittent catheterization, *K. pneumoniae* 33.3%, *P. mirabilis* 33.3%, *E. faecalis* 16.7%, and *K. oxytoca* 16.7%; in indwelling catheterization, *E. coli* 20.6%, *K. pneumoniae* 14.7%, *M. morganii* 14.7%, *P. aeruginosa* 14.7%, *E. faecalis* 11.8%, *P. mirabilis* 11.8%, *K. aerogenes* 5.9%, *E. cloacae* 2.9%, and *S. marcescens* 2.9%; in suprapubic catheterization, *Candida* spp. 50% and *P. aeruginosa* 50%; in condom catheterization, *E. coli* 66.7% and *E. faecalis* 33.3%; and in spontaneous voiding, *E. coli* 45.4%, *K. pneumoniae* 27.3%, *E. faecalis* 9.1%, *P. mirabilis* 9.1%, and *S. marcescens* 9.1%, as summarized in Table 3.

**Table 3:** Microorganisms by the voiding method in UTCs.

	Intermittent self-catheterization		Intermittent catheterization		Indwelling catheterization		Suprapubic catheterization		Condom catheterization		Spontaneous voiding		Total	
	N	%	N	%	N	%	N	%	N	%	N	%	N	%
*Candida* spp.							1	50.0%					1	1.0%
*Citrobacter koseri*	1	2.2%											1	1.0%
*Citrobacter freundii*	1	2.2%											1	1.0%
*Klebsiella aerogenes*	1	2.2%			2	5.9%							3	3.0%
*Enterobacter cloacae complex*					1	2.9%							1	1.0%
*Escherichia coli*	24	53.3%			7	20.6%			2	66.7%	5	45.4%	38	37.6%
*Klebsiella oxytoca*	1	2.2%	1	16.7%									2	2.0%
*Klebsiella pneumoniae ssp pneumonia*	13	29%	2	33.3%	5	14.7%					3	27.3%	23	22.8%
*Morganella morganii ssp morganii*	1	2.2%			5	14.7%							6	5.9%
*Proteus mirabilis*			2	33.3%	4	11.8%					1	9.1%	7	6.9%
*Pseudomonas aeruginosa*	2	4.5%			5	14.7%	1	50.0%					8	7.9%
*Serratia marcescens*					1	2.9%					1	9.1%	2	2.0%
*Streptococcus agalactiae*	1	2.2%											1	1.0%
*Enterococcus faecalis*			1	16.7%	4	11.8%			1	33.3%	1	9.1%	7	6.9%
													101	100.0%

N, number.

For the 5 most frequent bacteria, an antibiotic susceptibility profile was determined according to the latest EUCAST guidelines and organized as given in Table 4. Percentages shown include isolates belonging to the categories susceptible with the standard dosing regimen (S) and susceptible with increased exposure (I), as defined in the EUCAST guidelines. For *P. aeruginosa*, as a consequence of the breakpoints defined by EUCAST guidelines (starting from version 10.0)^7^ for ceftazidime, piperacillin/tazobactam, ciprofloxacin, and levofloxacin, all isolates not considered resistant to these antibiotics are considered to belong to category I. Blank spaces indicate either natural resistance or the insufficient number of isolates tested.

**Table 4:** Antibiotic susceptibility profile.

	Amikacin	Amoxicillin/clavulanic acid	Ampicillin = Amoxicillin	Cefepime	Cefotaxime	Ceftazidime	Ceftriaxone	Cefuroxime	Ciprofloxacin	Colistin	Ertapenem	Fosfomycin	Gentamicin	Levofloxacin	Linezolid	Meropenem	Nitrofurantoin	Piperacillin/tazobactam	Tigecycline	Trimethoprim-sulfamethoxazole	Vancomycin
*Escherichia coli* (N = 93)	100%	54%	42%	92%	85%	87%	74%	75%	60%	100%	100%	98%	89%	27%		100%	99%	97%		63%	
*Klebsiella pneumoniae ssp pneumoniae* (N = 44)	100%	75%		89%	80%	80%	79%	79%	77%	100%	100%		91%			100%		86%		77%	
*Proteus mirabilis* (N = 18)	100%	78%	22%	100%	100%	100%	100%	100%	39%		100%		72%	11%		100%		100%		28%	
*Pseudomonas aeruginosa* (N = 23)	74%			100%		91%			48%	100%				43%		100%		78%			
*Enterococcus faecalis* (N = 16)		100%	100%						56%					56%	100%		100%	100%	100%		100%

N, number.

Concerning specific multidrug-resistant organisms (MDROs, identified in 11.5% of isolates [27]), methicillin-resistant *S. aureus* (MRSA) was identified in 75% (3) of isolates of *S. aureus*; extended-spectrum beta-lactamases (ESBLs) were present in 9.8% (23) of total urine isolates, in 15.4% (14) of *E. coli* isolates, and in 20.9% (9) of *K. pneumoniae* isolates; there were multiresistant *P. aeruginosa* in 4.3% (1) of the total *P. aeruginosa* isolates; there were no carbapenem-resistant Enterobacterales (CRE). 51.9% (14) of isolated MDROs were identified in patients with indwelling catheters, 33.3% (9) were identified in patients with intermittent self-catheterization, 11.1% (3) were identified in patients with spontaneous voiding, and 3.7% (1) were identified in patients with suprapubic catheterization.

**Figure 3.**
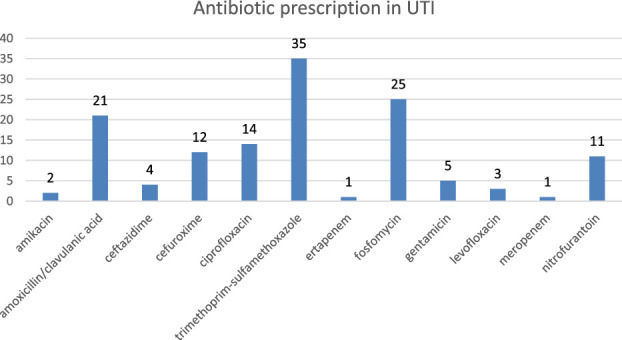
Antibiotics prescribed in UTIs.

Antibiotic prescriptions in UTIs were according to antibiograms, as shown in Figure 3: trimethoprim-sulfamethoxazole, 35 (25%); fosfomycin, 25 (19%); amoxicillin/clavulanic acid, 21 (16%); ciprofloxacin, 14 (11%); cefuroxime, 12 (9%); nitrofurantoin, 11 (8%); gentamicin, 5 (4%); ceftazidime, 4 (3%); levofloxacin, 3 (2%); amikacin, 2 (1%); ertapenem, 1 (1%); and meropenem, 1 (1%).

## Discussion

Overall, *E. coli* was the most frequent bacterium (finding in line with literature^1^), followed by *K. pneumoniae*.

88.4% of isolates were gram-negative bacteria. This type of pathogens is most commonly isolated from urine samples.^1^

Microorganisms identified in both UTIs and UTCs are distinct on different types of voiding methods.

Patients with an indwelling catheter, combined with patients with intermittent self-catheterization (which, together, are 62.1% of total patients) account for 72.3% of total isolates and 85.2% of MDRO isolates. Catheterization seems to be a risk factor of bacteriuria and the occurrence of drug resistance.

Urinary catheters (both intermittent and indwelling) predispose to catheter-associated urinary tract infections (CA-UTIs), biofilm formation, and encrustation.^8,9^ CA-UTIs seem to be due to external introduced contamination and internal microflora.^10^ In intermittent catheter users, there is a high risk of introduction of microflora of the meatus through the urethra up to the bladder; CA-UTIs are mainly caused by *E. coli*.^11^ In indwelling catheter users, the major problem is formation of bacterial biofilms; CA-UTIs are mainly caused by *P. mirabilis*.^12^ In this study, CA-UTIs (both intermittent and indwelling catheters) were mainly caused by *E. coli*.

CA-UTI microorganisms are more often resistant to usually recommended empirical antibiotics than non–CA-UTI microorganisms.^13^

In PRM outpatient consultation of the district Portuguese hospital, physiatrists require urine cultures both routinely (with other examinations) and when patients with SCI complain of new onset of signs and/or symptoms suggesting UTIs. They usually wait for urine culture results (microorganism identification and antimicrobial susceptibility testing) to then start directed antibiotic therapy in UTIs (avoiding empirical antibiotic therapy and consequent spread of resistant microorganisms). This antibiotic prescription in UTIs according to antibiograms seems to prevent development of MDROs. In fact, the European Association of Urology states that a “urine specimen for culture should be obtained prior to initiating antimicrobial therapy for presumed CA-UTI due to the wide spectrum of potential infecting organisms and the increased likelihood of antimicrobial resistance” and defends that the choice of antibiotics generally should be guided by severity of illness, the local antibiotic resistance data (antibiogram), host factors (including allergies), and sensitivity data of the isolated microorganisms.^1^ Recent studies also emphasize the importance of urine sampling for culturing before initiating antibiotic therapy for CA-UTIs.^13^

Symptomatic CA-UTIs should be treated according to the recommendations for complicated UTIs, as causative microorganisms in CA-UTIs are comparable with causative microorganisms in other complicated UTIs.^14^

The 2 most frequently prescribed antibiotics (trimethoprim-sulfamethoxazole 25% and fosfomycin 19%) were normal first-line antibiotics for noncomplicated UTIs (i.e., nitrofurantoin, trimethoprim-sulfamethoxazole, and fosfomycin).

For the 5 more frequent bacteria, the antibiotic susceptibility profile was determined. There seemed to be high resistance to fluoroquinolones: aside from resistance of *K. pneumoniae* to ciprofloxacin, all species had 40% or more of tested isolates resistant to both ciprofloxacin and levofloxacin. On the contrary, regarding cephalosporins, all 5 species had 26% or less of tested isolates resistant to any drug of this class. Of note, resistance to nitrofurantoin of *E. coli* and *E. faecalis* is very low; this antibiotic is considered first-line for uncomplicated cystitis caused by these agents and is administered orally, which is an advantage over, e.g., most cephalosporins and all aminoglycosides.

This antibiotic resistance profile (very low resistance to nitrofurantoin of 2 selected bacteria, low resistance to cephalosporins, and high resistance to fluoroquinolones) can, in selected cases, facilitate empiric antibiotic selection in patients with SCI.

This study has limitations: the sample is relatively small; it includes only patients with SCI followed in PRM outpatient consultation (which may have introduced a selection bias).

This study, through the characterization of the microbiological profile of urine cultures in patients with SCI and determination of the antibiotic susceptibility profile of the most common microorganisms, made it possible to track antibiotic resistance and facilitate empiric antibiotic selection.

## References

[1] Kranz J Bartoletti R Bruyère F . European association of Urology guidelines on urological infections: summary of the 2024 guidelines. Eur Urol. 2024;86:27–41. 38714379 10.1016/j.eururo.2024.03.035

[2] The prevention and management of urinary tract infections among people with spinal cord injuries. National Institute on Disability and Rehabilitation Research Consensus Statement. January 27-29, 1992. J Am Paraplegia Soc. 1992;15:194–204. 1500945 10.1080/01952307.1992.11735873

[3] Goetz LL Cardenas DD Kennelly M . International spinal cord injury urinary tract infection basic data set. Spinal Cord. 2013;51:700–704. 23896666 10.1038/sc.2013.72

[4] Kouri T Hofmann W Falbo R . The EFLM European Urinalysis guideline 2023. Clin Chem Lab Med. 2024;62:1653–1786. 38534005 10.1515/cclm-2024-0070

[5] Truong WR Hidayat L Bolaris MA Nguyen L Yamaki J. The antibiogram: key considerations for its development and utilization. JAC Antimicrob Resist. 2021;3:dlab060–6. 34223122 10.1093/jacamr/dlab060 PMC8210055

[6] The European Committee on Antimicrobial Susceptibility Testing. Breakpoint tables for interpretation of MICs and zone diameters. Version 14.0, 2024. Available at: http://www.eucast.org. Accessed July 24, 2024

[7] The European Committee on Antimicrobial Susceptibility Testing. Breakpoint tables for interpretation of MICs and zone diameters. Version 10.0, 2020. Available at: http://www.eucast.org. Accessed July 24, 2024

[8] Cortese YJ Wagner VE Tierney M Devine D Fogarty A. Review of catheter-associated urinary tract infections and in vitro urinary tract models. J Healthc Eng. 2018;2018:2986742. 30405898 10.1155/2018/2986742 PMC6204192

[9] Shahriar A Rob Siddiquee MF Ahmed H . Catheter-associated urinary tract infections: etiological analysis, biofilm formation, antibiotic resistance, and a novel therapeutic era of phage. Int J One Health. 2022;8:86–100.

[10] Whiteside SA Razvi H Dave S Reid G Burton JP. The microbiome of the urinary tract—a role beyond infection. Nat Rev Urol. 2015;12:81–90. 25600098 10.1038/nrurol.2014.361

[11] Nicolle LE. Catheter associated urinary tract infections. Antimicrob Resist Infect Control. 2014;3:23. [online serial]. 25075308 10.1186/2047-2994-3-23 PMC4114799

[12] Norsworthy AN Pearson MM. From catheter to kidney stone: the uropathogenic lifestyle of Proteus mirabilis. Trends Microbiol. 2017;25:304–315. 28017513 10.1016/j.tim.2016.11.015 PMC5365347

[13] D'Incau S Atkinson A Leitner L . Bacterial species and antimicrobial resistance differ between catheter and non-catheter-associated urinary tract infections: data from a national surveillance network. Antimicrob Steward Healthc Epidemiol [Online Serial]. 2023;3:e55. 36970431 10.1017/ash.2022.340 PMC10031580

[14] Cek M Tandoğdu Z Wagenlehner F Tenke P Naber K Bjerklund-Johansen TE. Healthcare-associated urinary tract infections in hospitalized urological patients-a global perspective: results from the GPIU studies 2003-2010. World J Urol. 2014;32:1587–1594. 24452449 10.1007/s00345-013-1218-9

